# Understanding power in food policy: a critical scoping review of methods to guide future research

**DOI:** 10.1093/heapro/daag104

**Published:** 2026-07-28

**Authors:** Naomi Fallon, Megan Romania, Christopher Yap, Christina Vogel

**Affiliations:** Centre for Food Policy, School of Health and Medical Sciences, City St George’s, University of London, Northampton Square, London, EC1V 0HB, United Kingdom; Centre for Food Policy, School of Health and Medical Sciences, City St George’s, University of London, Northampton Square, London, EC1V 0HB, United Kingdom; Centre for Food Policy, School of Health and Medical Sciences, City St George’s, University of London, Northampton Square, London, EC1V 0HB, United Kingdom; Centre for Food Policy, School of Health and Medical Sciences, City St George’s, University of London, Northampton Square, London, EC1V 0HB, United Kingdom; Medical Research Centre Lifecourse Epidemiology Centre, University of Southampton, Tremona Road, Southampton, SO16 6YD, United Kingdom

**Keywords:** power, food policy, food systems, review, research methods

## Abstract

Power disparities in the food system are reflected in food policy processes and outcomes that are failing to address major food system challenges. As interest in investigating and addressing these power disparities grows, it is important to assess the research methods that have been used to examine power in food policy, and how methodological choices shape our understanding. This scoping review critically maps the methodological approaches applied in empirical studies of power in national-level food policy. A systematic search across fourteen databases identified 46 empirical studies published between 2014 and 2025: 33 qualitative, 9 discourse analytic and 4 quantitative. Qualitative research examined the relative power of food system actors and their strategies, as well as the structural dynamics sustaining unequal influence. Quantitative analyses studied the patterns of structural and positional power within policy networks and across political systems. Discourse analytic studies examined how ideas and discourses construct and legitimize understandings of food policy problems and solutions. Across methods, most research investigates how entrenched power constrains progress on food system challenges, with fewer studies exploring examples of resistance. Each approach demonstrates strengths and limitations that are shaped by theory and data. This review highlights the need for methodological innovation—including applying mixed methods, longitudinal designs, and comparative analyses—to expand our understanding of power in food policy and identify actionable strategies to address disparities for the benefit of people and planet.

Contribution to Health Promotion

- This review shows the wide range of ways researchers study power in food policy, and how these shape our understanding.
- Most studies use qualitative methods, whilst quantitative and discourse-based approaches are used less often.
- Current research mainly highlights how powerful actors block progress, with less focus on how change can succeed.
- Using mixed, longitudinal, and comparative methods could improve understanding of how power works and support healthier, more sustainable food policies.

## Introduction

Countries around the world are facing critical food system challenges that impact human and planetary health (Swinburn *et al*. 2019). With many of the policies needed at the country level, national governments have a critical role in driving through integrated policies that enable the transition towards healthy and sustainable diets (Temme *et al*. 2020). However, evidence that powerful actors have delayed and diluted previous policy efforts to improve public health (Knai *et al*. 2015) highlight how power disparities in the food system lead to food policy processes and outcomes that fail to achieve societal goals (Lacy-Nichols and Marten 2021, Slater *et al*. 2022). In this context, scholars have called for greater critical, empirical attention to power in food policy (Lacy-Nichols and Marten 2021, Walls *et al*. 2021, Clapp *et al*. 2025).

In recent years, scholars have responded to these calls, examining power in food policy through a range of study designs and research methods, including case studies (Phulkerd *et al*. 2022), social network analyses (Cullerton *et al*. 2016), and critical discourse analyses (CDA) (Omar and Thorsøe 2024). These studies are shaped by a variety of different theoretical and analytical orientations. Studies frequently adopt a relational view of power, influenced most commonly by Lukes’ (1974) three faces of power. These studies examine how actors such as corporations, governments, and civil society mobilize different forms of power across policy spaces (Clapp *et al*. 2009, Barlow and Thow 2021). Other studies draw from structural perspectives of power, including political economy approaches, to scrutinize the economic structures, class relations and institutional arrangements driving food policy processes and outcomes (Walls *et al*. 2021, Voigt *et al*. 2024). A third group of studies focus on how power manifests and is mobilized through discourse and ideas (Omar and Thorsøe 2024). Taken together, these varied approaches reflect a recognition that power is not only exercised by visible actors, but also embedded in institutions, systems, and discourses. Public policy and health policy scholarship has highlighted methodological challenges in analysing power, including in food policy contexts (Buse *et al*. 2023). Yet, despite this growing body of work, there has been limited reflection on how different research methods have been applied in food policy research to date, and how they shape our understanding of power in this emerging field.

Several reviews have consolidated evidence on how powerful actors, particularly corporations, shape food systems, and food policy (Wood *et al*. 2021; Chavez-Ugalde *et al*. 2021). These reviews focus primarily on what the studies show without examining the methodological choices that underpin this knowledge. Other reviews have centred the conceptual (Sodano and Gorgitano 2022) and analytical (Walls *et al*. 2021) approaches to studying power. These contributions have highlighted persistent challenges, including significant ‘conceptual heterogeneity’ (Sodano and Gorgitano 2022) and the need for more theoretically informed and methodologically robust research (Walls *et al*. 2021). Yet, across both strands, there has been little attention to how research methods are used in combination with theory and analytical frameworks to study power in food policy. Addressing this gap is crucial for identifying how different research methods can be used to examine the different dynamics of power in food policy and to identify opportunities for intervention.

This critical scoping review aims to address this evidence gap by systematically mapping the methodological landscape of studies investigating power in national-level food policy. The findings of this review will provide guidance on appropriate methods for future research to strengthen the evidence base for understanding existing power dynamics in food policy and identifying where and how power disparities in food policy can be minimized for the benefit of human and planetary health. This critical scoping review therefore addressed the following research questions:

1. What methodological approaches have been used to study power in national-level food policy?
2. How does analysing power through different methodological lenses shape our understanding of the power dynamics in food policy?

## Methodology

This study adopted a scoping review study design following the Joanna Briggs Institute protocol (Peters *et al*. 2020, Aromataris *et al*. 2024) and reported using the PRISMA Extension for Scoping Reviews PRISMA-ScR checklist (Tricco *et al*. 2018) (see Supplementary File S1). A scoping review was selected over other types of review due to its suitability for mapping diverse literature, including multiple methodological approaches (Peters *et al*. 2020) and study designs (Khalil *et al*. 2022). A research protocol was uploaded to Open Science Framework (Fallon *et al*. 2024).

### Search strategy and selection criteria

The search strategy and selection criteria were developed using the Population, Concept, Context framework (Aromataris *et al*. 2024). The population included stakeholders involved in national-level policymaking from across the food system, including (i) policymakers and other public sector stakeholders, (ii) market actors, (iii) third sector actors, and (iv) community actors (Avelino and Wittmayer 2016).

The concept included any social science conceptualization and definition of power. Synonyms such as ‘influence’, ‘agency’, and ‘authority’ were excluded to maintain conceptual specificity and to generate a manageable sample size. To reflect the methodological focus of the study, only studies that stipulated power as an intended outcome or predictor variable were included. Studies where power emerged as a finding or was limited to the introduction or discussion sections were excluded.

The context focused exclusively at the national-level public food policy setting. Studies of local, global, and multi-level policy and governance were excluded to enable the production of practical guidance given the significant heterogeneity of the sample. Here, ‘food policy’ is defined as public policies developed and enacted by governmental authorities and associated agencies (Wells *et al*. 2025). Food policy includes policies from different ‘policy domains’ (Burstein 1991) including nutrition and health, agriculture, food safety, food security, trade, environmental sustainability, and animal welfare. Analyses of health policy or non-communicable disease (NCD) policy were included only if they concerned food-related products, activities, or issues. Similarly, climate policy was included only if the study explicitly concerned food-related agricultural activities.

The search strategy was trialled and refined in an iterative process (Dixon-Woods *et al*. 2006). A full list of all search terms used in database searches is available in Supplementary File S2. Fourteen databases were systematically searched in June 2024, and again in June 2025, by the first author using four platforms (EBSCO Host, Scopus, Web of Science, and Ovid Online). Searches were limited to English language studies published in peer-reviewed journals on or after 2014 to reflect recent research covering the last decade. Empirical research studies of all research designs, including both qualitative and quantitative studies, were considered.

### Screening

Screening was conducted by two authors using a pragmatic approach to balance the need for rigour with resource constraints (Mak and Thomas 2022). First, two authors independently screened 20% of the sample to assess consistency in decision-making and refine the selection criteria (Mak and Thomas 2022). The first author then screened all remaining texts, and the second author screened 50% in total. Any discrepancies were resolved through discussion between the two authors. The process was repeated for the full text screening. At all stages, the level of disagreement between authors was below the 10% permitted in this approach (Mak and Thomas 2022). In line with common practice for scoping reviews (Peters *et al*. 2020), quality assessment on individual studies was not conducted—studies were included based on relevance to the research questions.

### Data extraction and synthesis

Data were extracted into a bespoke database developed by the first author and refined after piloting by two authors. Data were extracted by the first author for study characteristics (author and year, publication, country/countries, study population, and policy topic), research methods (research design, data collection methods, data analysis methods, and conceptual/theoretical/analytical framework), methodological strengths and limitations, key findings, and recommendations for future research. Categorization decisions were reviewed and refined through discussion with all authors, which provided a consistency check for studies where the policy focus or methodological features were less clear-cut. Although extraction was not formally duplicated, this collaborative review process ensured coherence and reduced subjectivity in the final classifications.

Synthesis was conducted by the first author. Preliminary findings were discussed with all authors to increase validity and reduce subjectivity in the conclusions were drawn. To address research question 1, the study design characteristics and research methods were charted and a descriptive narrative summary produced. To address research question 2, principles from critical interpretive synthesis (CIS) were used to analyse the methodologically varied literature (Dixon-Woods *et al*. 2006). CIS enabled a critical assessment of the existing evidence base beyond the descriptive summaries typical in scoping reviews (Dixon-Woods *et al*. 2006). Differences and contradictions in evidence and theory across the studies were examined, and the strengths and limitations of methodologies used were critically assessed and presented in the discussion to inform future research design (Dixon-Woods *et al*. 2006). This approach has been used in related research, including analyses of the commercial determinants of dietary behaviour (Chavez-Ugalde *et al*. 2021).

Methodological design was organized into three categories—qualitative, quantitative, and discourse analytic—to support a clear synthesis of diverse approaches. Discourse analytical studies were grouped separately to reflect their shared focus on language-in-use, and use of specific methods and theoretical orientations (Wetherell *et al*. 2001). Whilst discourse analysis is situated within qualitative research traditions, the emphasis on the role of language in constructing social realities sets it apart from other qualitative approaches (Wodak and Meyer 2016), making it a useful distinction to guide scholars in future research design.

The policy focus of studies was also organized into three categories: policy process, policy documents, or policy domain. The policy process category includes studies that examine how food policies are developed. The policy document category covers studies that analyse the content of specific policy texts. The policy domain category includes studies that take a broader perspective, exploring the power dynamics within a policy area (e.g. nutrition policy), without focusing on a specific policy. Although some studies engaged with more than that one aspect of policy (e.g. drawing on policy documents as part of an examination of a policy process), categorization was based on the study’s analytical focus, reflecting the main object of analysis, and level of abstraction. Each study was therefore assigned to a single category.

## Results

### Study characteristics of included articles

**Figure 1:**
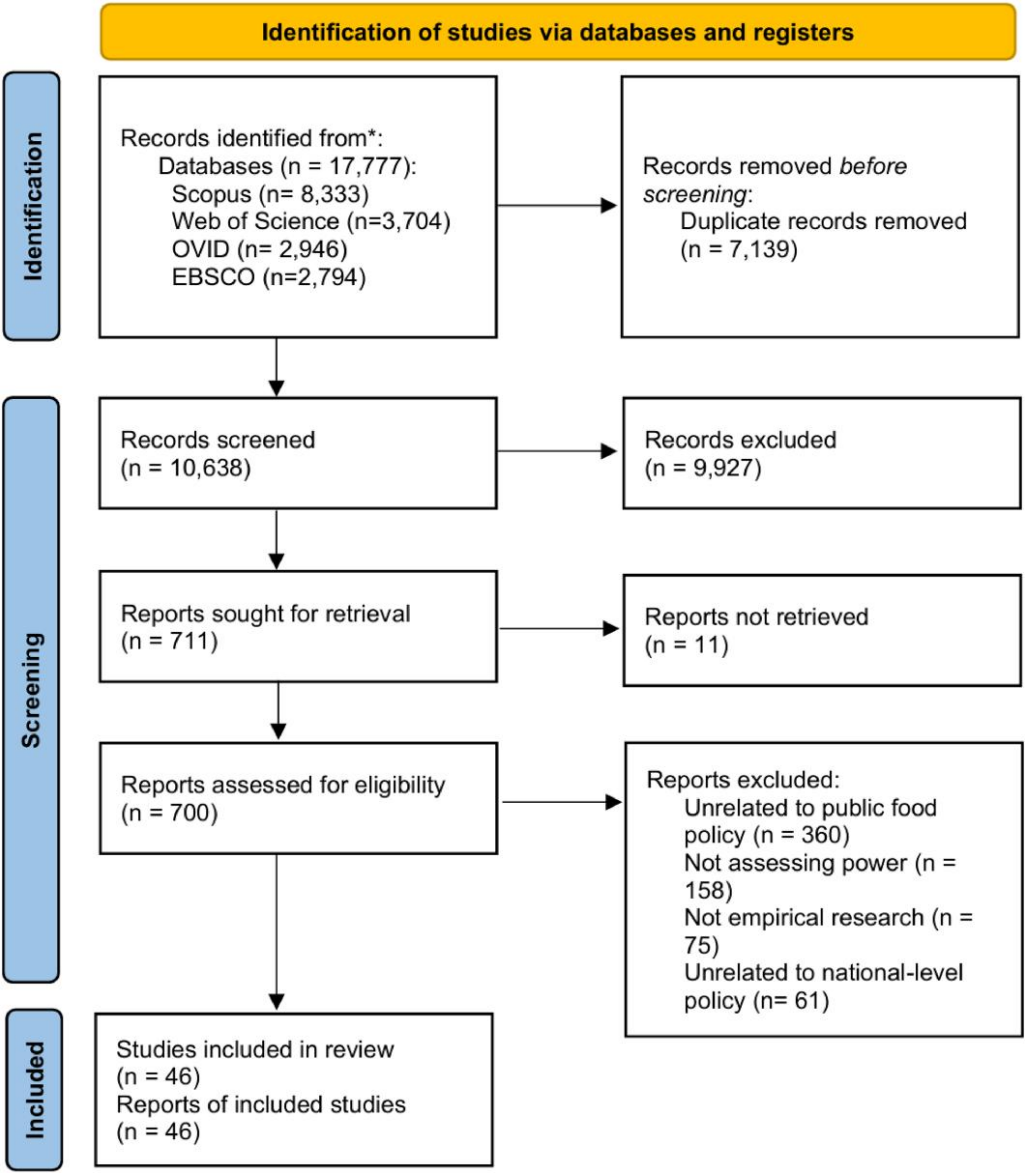
PRISMA-flow diagram of the search and screening process (Tricco *et al*. 2018). Database searches identified 10 638 records after de-duplication. After title and abstract screening, 9927 studies were excluded, and the full text was unavailable in a further 11, leaving 700 for full text screening. Of these, 46 studies met the inclusion criteria and were included in the scoping review.

In total, 10 638 articles were identified through the search and screened at the title and abstract stage. A total of 700 were screened at the full text stage and 46 studies met the inclusion criteria for analysis (Supplementary File S3). See Fig. 1 for full search and screening results.

The number of studies published between 2014 and 2024 were variable. No eligible studies were published in 2014 or 2015. Most studies (61%) were published in the past 5 years (2021–5), signalling an increase in research interest.

Most studies focused on a single country context (*n* = 43). The other studies assessed three (*n* = 2) and 56 (*n* = 1) countries. Aside from this latter study, which did not disaggregate country-specific data, 49 countries were studied. Studies were spread across the continents: Africa (*n* = 13), Europe (*n* = 12), Asia (*n* = 6), Oceana (*n* = 10), and the Americas (*n* = 8). Australia was the most studied country (*n* = 8), followed by the UK (*n* = 3). Using World Bank Classification system, most studies were of high-income countries (HIC) (*n* = 25), followed by lower middle-income countries (*n* = 9). Fewer studies assessed power in upper middle-income countries (*n* = 8) and low-income countries (*n* = 7) (The World Bank n.d).

**Figure 2:**
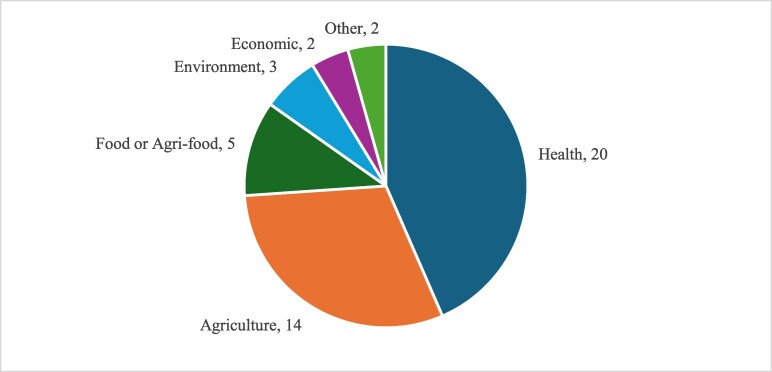
Distribution of included studies across policy domains. Health (20) and agriculture (14) comprised the largest share, followed by food or agri-food (5), environment (3), economic (2), and other (2) policy domains.

Figure 2 displays the policy domain of included studies. Topics related to health (*n* = 20) and agriculture (*n* = 14) policy domains were by far the most studied across the sample, covering topics including sugar-sweetened beverage (SSB) taxes (*n* = 4), food labelling regulation (*n* = 5), food marketing regulation (*n* = 4), and particular agriculture strategies or Bills (*n* = 4). Studies also examined national-level food or agri-food strategy (*n* = 5), and topics related to environmental (*n* = 3), and economic (*n* = 2) policy domains. The study sample included studies that examined the development process of specific policies (*n* = 28), policy documents (*n* = 8), and power dynamics in the broader policy domain (*n* = 10).

### Methodological approaches

A range of methods were used in the included studies, which we categorized according to three types of methodological approaches: qualitative, discourse analytic, and quantitative. Although discourse analytic studies sit within qualitative research traditions, they were reported separately here to reflect their distinct focus on how language frames policy problems and positions actors, revealing how power works through discourse. Most of the studies used qualitative (*n* = 33) study designs, compared with discourse analytic (*n* = 9) and quantitative (*n* = 4) approaches (Table 1). The studies drew from a wide range of power theories (*n* = 8) and analytical frameworks (*n* = 13) to guide their data analysis.

**Table 1:** Methodological characteristics of included studies, including the number of qualitative, quantitative and discourse analytic studies, and their most common study types and data sources.

Methodological approach	Study types	Data sources	Studies
**Qualitative (*n* = 33)**	Qualitative Case study Ethnography	Interview (*n* = 28) Documents (*n* = 24) Observations (*n* = 6) Focus groups (*n* = 3) Ethnographic fieldwork (*n* = 1) Workshops (*n* = 1)	Alpha and Fouilleux (2018), Autzen and Hegland (2021), Babu and Akramov (2022), Baker *et al.* (2017), Browne *et al.* (2021), Carey *et al.* (2017), Carriedo *et al.* (2020), Chinsinga and Chasukwa (2018), Coulas (2021), Elliott *et al.* (2023), Fialon *et al.* (2022), Fisher *et al.* (2021), Georgekutty and Varghese (2024), Gómez-Dantes *et al.* (2021), Harris (2019a, 2019b), Hernandez (2017), Kapetanaki *et al.* (2021), Kinniburgh (2023), Madurawala *et al.* (2023), Milsom *et al.* (2023), Moyo *et al.* (2025), Phulkerd *et al.* (2022), Pomeranz *et al.* (2020), Sackar *et al.* (2023), Sing *et al.* (2025), Thow *et al.* (2021), Van Lieshout *et al.* (2017), Venegas Hargous *et al.* (2025); Voigt *et al.* (2024), Wahnschafft *et al.* (2024), Yami *et al.* (2019), Zinsli (2023)
**Discourse analytic (*n* = 9)**	CDA, Foucauldian analysis, WPR	Documents (*n* = 9) Interviews (*n* = 2)	Andersson *et al.* (2022), Attorp and McAreavey (2020), Brookes (2021), Larsson and Vik (2023), Mathez and Loftus (2023), McCartan *et al.* (2025), O'Keeffe (2017), Ralston *et al.* (2023), Tourangeau (2018)
**Quantitative (*n* = 4)**	Observational Cross-sectional Comparative Longitudinal	Surveys (*n* = 3) Datasets (*n* = 1)	Cullerton *et al.* (2016), Henning *et al.* (2019), Moschitz *et al.* (2016), Thomson (2017)

#### Qualitative approaches

In total, 33 studies employed qualitative empirical methods to analyse power in food policy. Of these, 13 studies adopted a case study research design, comprising single within-case (*n* = 11) and multiple case (*n* = 2) study designs. The studies drew from a range of data sources, including interviews (*n* = 28), documentary sources (*n* = 24), observations (*n* = 6), focus groups (*n* = 3), ethnographic fieldwork (*n* = 1), and workshops (*n* = 1). Most studies employed more than one method of data collection (*n* = 22), whereas 11 studies employed a single method, most commonly interviews (*n* = 8).

The most common forms of analysis were thematic analysis (*n* = 15), policy analysis (*n* = 5), content analysis (*n* = 5), and stakeholder analysis (*n* = 3). Power mapping tools PolicyMaker (Reich and Campos 2020) (*n* = 1) and NetMap (Schiffer and Hauck 2010) (*n* = 1), and systems dynamic modelling (*n* = 1) were used to rank or map power dynamics between actors using qualitative data.

A range of theoretical and analytical frameworks were used to facilitate the analysis involving different conceptualizations and categorizations of power (Table 2). Seven studies used multi-dimensional frameworks that build on Lukes’ (1974) three faces of power, including the corporate power framework (Fuchs and Lederer 2007) (*n* = 3), Power Cube (Gaventa 2006) (*n* = 2), and Milsom’s *et al*. (2021) framework (*n* = 1). Lukes’ theory distinguishes between *decision-making power*—an actor’s capacity to directly influence policy formation; *non-decision-making power*—the ability of an actor to shape the agenda and behaviours of others indirectly; and *ideological power*—the ability to influence public and political debate, to shape how policy issues are understood, and which policies are accepted as normal or socially desirable. These studies examined how different actors mobilized different forms of power across policy spaces, such as in food marketing policy in Thailand (Phulkerd *et al*. 2022).

**Table 2:** Analytical frameworks used in qualitative studies to examine power, including their key analytical categories and associated author(s).

Framework	Categories	Author(s)
**Corporate power framework**	Structural, instrumental, and discursive forms of power	Fuchs and Lederer (2007)
**Power** c**ube**	Forms, levels and spaces of power	Gaventa (2006)
**Power in public health policymaking framework**	Forms, mechanisms, dimensions, and outcomes of power	Milsom *et al*. (2021)
**3I’s political economy framework**	Interests, ideas, institutions	Hall (1997)
**Ideational power framework**	Power in ideas, power through ideas, power over ideas	Carstensen and Schmidt (2016)
**Power in interactive governance**	Power over, power in, power of’ governance processes	Torfing *et al*. (2012)
**Power in planning and policy framework**	Relational power, dispositional power, structural power	Arts and Tatenhove (2004)
**Systematization of power resources**	Organizational, discursive, systemic, strategic-structural selectivities	Buckel *et al*. (2014)

Five studies used approaches from political economy. Political economy approaches are concerned with how circulations between the social, economic, environmental, and governance dimensions of society shape power relations (Dow 2023, Madurawala *et al*. 2023). Three studies used the 3I’s framework to frame their analysis through three features of policy development processes: *interests*, *ideas*, and *institutions* (Hall 1997). Some political economy studies combined political economy frameworks with other critical, sociological, and policy theories to facilitate their analysis of structural aspects of power. For example, Elliott *et al*. (2023) drew from Giddens’ (1984) structuration theory, Bourdieu’s (1986) forms of capital, and post-colonial theory of epistemic injustice (Bhakuni and Abimbola 2021) in their analysis of the systemic power imbalances that shaped a SSB tax in Vanuatu.

Three studies drew from new institutionalism to examine the formal and informal ‘rules, norms, practices, and relationships that influence patterns of behaviour in politics and policymaking’ (Cairney 2019, p. 75). Of these, two studies used the ideational power framework, which delineates between three aspects of ideational power: the institutionalization of ideas (*power in ideas*), the content of the ideas as a means to persuade (*power through ideas*), and the process through which ideas are communicated (*power over ideas*) (Carstensen and Schmidt 2016).

Other frameworks used in qualitative studies included the power in planning and policy framework (Arts and Tatenhove 2004) (*n* = 1), which assesses actor power in terms of their ability to influence others (relational power), capacity to act based on resources or position (dispositional power), and power which is granted by systems and structures (structural power). In another example, Van Lieshout *et al*. (2017) combined a pragmatic conceptualization of power (Allen 2008) with the analytical framework of ‘power over’, ‘power in’, and ‘power of’ governance processes (Torfing *et al*. 2012) to analyse the power dynamics between actors in agriculture policy discussions.

#### Discourse analytic approaches

Eleven studies employed discourse analytic methods to analyse how power manifests and is exercised in food policy. Documents were the primary data source used for these studies (*n* = 9), with sample sizes ranging from 1 to 108 documents. A sub-set of studies did not detail the specific documents included in the sample (*n* = 2), but these studies did supplement documentary sources with data from semi-structured interviews.

Studies drew from a range of theoretical and analytical approaches that have been grouped into the following: critical and argumentative discourse analysis (*n* = 5), Foucauldian (*n* = 2), and problem representation (*n* = 2).

Four studies conducted CDA (Tourangeau 2018, Attorp and McAreavey 2020, Brookes 2021, McCartan 2024). In CDA, language both shapes and is shaped by societal power relations, and as such the close examination of language can reveal the operation of power in society (Fairclough 2015). One study (Larsson and Vik 2023) used argumentative discourse analysis (Hajer 1995) to assess power through the storylines that dominated political discourse. Tourangeau (2018) employed CDA in combination with Fuchs and Lederer’s (2007) corporate power framework to understand the different ways that power manifests in parliamentary debates.

Two studies employed Foucauldian theory and approaches (O’Keeffe 2017, Mathez and Loftus 2023). Both drew on the concept of ‘governmentality’—the methods and tactics through which the state seeks to shape individual and collective conduct (Foucault 1982). For example, Mathez and Loftus (2023) examined how modernist development discourses can be reproduced through state mechanisms to inhibit action towards socio-ecological goals. O’Keeffe (2017) used a Foucauldian-inspired genealogical analysis—which examines the historical origins and evolution of discourse (Wetherell *et al*. 2001, Kearins and Hooper 2002)—to trace how responsibility over farming has shifted from the state towards private investment and markets.

Two studies (Andersson *et al*. 2022, Ralston *et al*. 2023) employed Bacchi and Goodwin’s (2016) What is the Problem Represented to Be? (WPR) approach, which highlights how dominant policy paradigms shape problem definitions and responses. For instance, Ralston *et al*. (2023) found that, despite political commitments to addressing structural drivers of obesity, policy document framed obesity as an issue of individual responsibility rather than commercial or environmental drivers.

#### Quantitative approaches

There were four quantitative studies comprising three network analyses and one cross-national regression analysis (see Table 2). These studies were mostly cross-sectional by design, offering a snapshot in a particular context and time. On occasion, analyses were extended through incorporating comparative (*n* = 2) and longitudinal (*n* = 1) elements.

Studies drew from different network analysis approaches; policy network analysis (*n* = 2) and social network analysis (*n* = 1), using data from quantitative surveys and interviews to examine the structure of a social (or policy) network using statistical modelling. The relations between actors identified who the most influential actors were based on their position and connections within that network (Rocker *et al*. 2022). For example, Moschitz *et al*. (2016) conducted a longitudinal policy network analysis using to analyse changes in actor relationships and influence in organic farming policy in the Czech Republic across two time points. These studies adopted a range of theoretical and analytical approaches to structure and facilitate their analysis. For example, Cullerton *et al*. (2016) drew on the advocacy coalition framework (Sabatier and Jenkins-Smith 1993) to structure their analysis of actor clusters in nutrition policy, whilst Henning *et al*. (2019) combined policy network theory and interest group theory to assess patterns of interaction and influence within participatory policy networks.

The cross-national observational study involved the analysis of data from multiple datasets using a fixed-effects panel regression model to estimate the relationship between political power structures and agricultural policy outcomes (Thomson 2017).

### How have different methods informed our understanding of the power dynamics in food policy?

Three ways were identified in which studies of power in food policy in this sample sought to extend understanding of power: (i) the power dynamics of and between *stakeholders*, and their strategies to influence policy, (ii) the *discursive and ideational* power dynamics embedded in, and mobilized through policy, and (iii) *structural* dynamics that shape policy to the benefit of some over others. Each methodological approach was found to have informed our understanding of these dynamics in distinct ways. A link was also identified between methodological choices and policy focus.

#### Actor dynamics

Qualitative and quantitative research methods offered distinct perspectives in the examination of stakeholder power dynamics. The qualitative studies offered contextualized insights on specific dynamics in policy processes, whereas the quantitative studies offered a macro-level perspective of the relative power of different stakeholders within a policy field or domain.

A variety of analytical approaches and frameworks were used to identify a few key actors—those who held the most and least power—within specific policy processes. For example, Gómez-Dantes *et al*. (2021) conducted a stakeholder analysis to ascertain the position and power of actors involved in policy discourse relating to a proposed SSB tax. They found that actors opposing an increase in the level of a SSB tax had greater political power than those supporting it, with strongest opposition coming from the food industry. Stakeholder mapping was also applied to the agricultural policy process in Tajikistan, where Babu and Akramov (2022) identified a concentration of power in central government that persisted across different development eras and structural/institutional changes (pre-1990’s Soviet, post-1990’s and post-1997). Qualitative analyses were also used to examine the different forms of power mobilized by different food system actors to influence policy. For example, Milsom *et al*. (2023) used qualitative system dynamics modelling to unpick the different mechanisms through which food industry actors exercised structural, instrumental, and discursive power over diet-related NCD policy in South Africa.

Whilst most studies focused on dominant food system actors (in particular food industry and state actors), some qualitative studies examined cases where less-powerful actors successfully overcame power imbalances to effect policy change. These studies identified four strategies: (i) building coalitions (*n* = 4), (ii) targeting advocacy efforts at powerful actors (*n* = 4), (iii) changing the narrative (*n* = 4), and (iv) building public support (*n* = 2) as successful strategies that could be mobilized by less-powerful actors to minimize power disparities in food policy making. Indeed, Wahnschafft *et al*. (2024) identified all four of these strategies as used by advocates in their successful push for the adoption of the ‘Promotion of Healthy Eating Law’ in Argentina in the face of corporate opposition.

In contrast to the qualitative studies, which examined specific processes, quantitative studies offered insights into the power dynamics within a particular policy domain. Quantitative network analysis techniques were used to quantify the power of different actors in a policy network (policy domain) based on their position in the network. For example, Moschitz *et al*. (2016) conducted a longitudinal policy network analysis to examine the power dynamics in a network based on changes in reputational power and the position of different actors over a 10-year period.

#### Structural dynamics

All three methodological approaches—qualitative, discourse analytic, and quantitative—were used to examine structural dynamics of power. However, overall, few studies within the sample focused on the structural dynamics of power. Qualitative methods informed by political economy and new institutionalism were used to analyse the systemic factors that uphold the dominance of specific actors or sectors and inform the exclusion and representation of marginalized groups. For example, Venegas Hargous *et al*. (2025) drew from critical theory to examine how people with lived experience of marginalization continued to experience systematic exclusion from nutrition policy processes and spaces. Several qualitative studies highlighted the contingent nature of structural power. For example, Voigt *et al*. (2024) highlighted how the dominant food regime were successfully challenged by emerging alternative ‘regimes’, underscoring that structural power can be resisted and reconfigured.

Discourse analytic methods were used to examine how structural power inequities are perpetuated through policy documents (Andersson *et al*. 2022, McCartan 2024). For example, McCartan (2024) analysed Aboriginal plant foods policy, revealing the colonial legacies and neoliberal ideologies that led to the exclusion of Indigenous actors from the policy process and the elevation of non-Indigenous knowledges and interests.

Whilst the discourse analytic and qualitative studies were often context specific, the single quantitative analysis of structural aspects of power offered cross-national insights into the political power of competing groups under different political structures (Thomson 2017). They used socio-economic measures of consumer and producer power as indicators of the relative political and economic power of rural and urban interests within the agricultural sector to explain the variation in level of agricultural support across democratic and authoritarian regimes.

#### Discursive and ideational dynamics

A range of discourse analytic and qualitative approaches were used to examine the dominant ideas and discourses in food policymaking, and their impacts. A consistent feature across the discourse analytic studies (*n* = 7) was a concern with neoliberal logics present in policy documents, including market liberalization and deregulation, and the emphasis on individual responsibility and consumer choice. These narratives were used to justify certain policy interventions, such as policies targeting individual behaviour change in obesity policy, despite acknowledgement of the causal role of systemic drivers (Brookes 2021, Ralston *et al*. 2023).

Two studies analysed how discourses of *post-exceptionalism* were mobilized in policy debates and documents to justify a shift in power away from primary producers to corporate actors (Attorp and McAreavey 2020) and to resist meat-reduction policies (Larsson and Vik 2023). Post-exceptionalism describes a move away from the special status (*exceptionalism*) afforded to farming interests in public policy towards a more integrated approach that includes consideration of environmental, societal, and economic concerns. However, both studies found that, in practice, economic and industry interests continued to dominate policy decisions.

Three qualitative studies examined the discursive and ideational dynamics of power. Each approached the analysis using theories from ‘new institutionalism’ to examine the relationship between institutions and ideas. For example, Coulas (2021) used the ideational power framework (Carstensen and Schmidt 2016) to show how the policy process to develop a national food policy was shaped by a combination of actor framings, historical institutional factors and ideas regarding the accepted remit of the policy. In addition, discursive and ideational power was examined to some extent as part of some multi-dimensional and political economy studies as one way power manifests and is mobilized in food policymaking.

Both qualitative and discourse analytic studies positioned ideas within their broader structural context. However, discourse analysis research also highlighted that the way issues are talked about not only reflects existing power dynamics, but also actively shapes them.

#### Power dynamics across different aspects of policy making

Each methodological approach had a different dominant policy focus (Table 3). Qualitative studies typically explored the context-specific dynamics of the policy development process (*n* = 26), showing how power dynamics influence policy design. Discourse analytic methods focused on the powerful narratives and structural power inequalities that are embedded in, and perpetuated through, policy documents (*n* = 7). Quantitative studies examined the power dynamics within broader policy domains (*n* = 3), identifying structural or systemic patterns within or across national contexts. This pattern suggests a mutually constitutive relationship between the selection of research methods, the aspect of policy making and how power is conceptualized and identified within food policy research.

**Table 3:** Policy focus of each methodological approach.

Methodological approach	Policy focus		
	Policy field (*n* = 10)	Policy document (*n* = 8)	Policy process (*n* = 28)
Qualitative (*n* = 33)	6	1	26
Discourse analytic (*n* = 9)	1	7	1
Quantitative (*n* = 4)	3	0	1

Qualitative studies most commonly examine policy process, discourse analytical studies focus on policy documents, and quantitative studies on the policy field level.

## Discussion

The findings of this study illustrate the diverse approaches to studying power in national-level food policy. Qualitative research dominates the field currently, whilst discourse analytic and quantitative studies remain underutilized. Each method and methodological approach offers distinct lenses that shape how we understand and characterize the dynamics of power in food policy.

Qualitative studies favoured a single-case study research design and using data from multiple sources, such as qualitative interviews, documents, and workshops. A range of conceptual and theoretical frameworks were used to guide the analysis and interpretation of different dynamics of power (Anfara and Mertz 2015, Collins and Stockton 2018). Discourse analytic methods—situated within qualitative research traditions but distinguished by their specific focus on language, meaning-making, and the discursive construction of social and political realities—were used to identify and critique dominant ideas present in policy documents and political discourse. In these studies, methods and theory were drawn from post-structuralist traditions, which foregrounds the constitutive role of language and discourse in shaping social reality (Foucault 1982, Fairclough 2015). Fewer studies used quantitative methods including network analyses and cross-national regression analysis to analyse the distribution of power in political systems and the relational and positional power of food systems actors within a policy domain.

The pattern observed between methodological approach and policy focus was unexpected. Each approach tended to concentrate on a different aspect of policymaking: qualitative studies primarily examine the policy process, discourse analytic research focused on policy documents, and quantitative studies tended to investigate at the policy domain level. It is unclear whether the observed clustering reflects the suitability of certain methods for examining particular aspects of policy, or whether it is a product of how the field has developed. Evidence from individual studies using quantitative (Henning *et al*. 2019) and discourse analytic studies (Tourangeau 2018) suggest that all three approaches can, for example, generate insights into the policy process.

Whilst the approaches differed in policy focus, they shared a dominant orientation towards diagnosing how entrenched power dynamics obstruct progress on societal challenges such as obesity, unsustainable food production, and food insecurity. This work reflects the burgeoning scholarship on the commercial determinants of health (Lacy-Nichols and Marten 2021) and corporate political activity (Mialon *et al*. 2020), which examine the problematic role of corporate actors in policymaking in food and other health-harming industries. Notably, only a small number of studies—all using qualitative methods—explored cases where less materially powerful actors succeeded in overcoming power disparities to influence policy outcomes. The strategies for actors seeking to influence food policy indicated from these studies were (i) building coalitions, (ii) targeting advocacy efforts at powerful actors, (iii) changing the narrative, and (iv) building public support, are supported by the broader literature (Friel 2020). However, other strategies in the literature were absent, including how institutional processes have been leveraged to disrupt power structures and achieve policy change (Friel 2020).

Each methodological approach exhibited strengths and limitations that should be considered when designing future research to study power in food policy. Qualitative case studies facilitated the in-depth exploration of a particular policy or policy process (Yin 2018), but a reliance on single-case designs limited the generalizability of findings (Elliott *et al*. 2023). Qualitative methods combined with theory provided rich insights into complex phenomena, but the use of prescriptive frameworks risks shaping findings in a way that limits the potential for unexpected discoveries (Collins and Stockton 2018). Theoretically informed discourse analytic research offered valuable insights into societal power dynamics, and the assumptions and ideas that informed policy choices. By interrogating how problems are framed, which voices are legitimized or silenced, and how particular narratives become normalized, discourse analytic research approaches illuminate dimensions of power that are less accessible through other qualitative methods. The focus on policy documents in these studies produced context-specific insights, but limited generalizability and did not shed light on the mobilization of power, and power struggles that occurred in the production of the policy document (Elliott *et al*. 2023). Finally, the reliance on publicly available documents means that potentially important interactions outside of public view may have been missed (Townsend *et al*. 2019). Quantitative methods helped identify broader patterns of power in policy domains and demonstrated the utility of incorporating comparative or longitudinal methods into research design. However, these studies offer a simplified snapshot of power and limited insights into the dynamic nature of policy making (Cullerton *et al*. 2016, Thomson 2017). Across the methods, there was a reliance on subjective assessments of power and publicly available documents, which were used to expose bias in the policy process, assumptions, and dominant framings. However, the sole reliance on such sources also risks bias or misunderstanding, especially with the recognized difficulty in engaging with corporate actors, high-level political figures, and marginalized groups (Carriedo *et al*. 2020, Fialon *et al*. 2022, Milsom *et al*. 2023).

The strengths of each method demonstrate the value of methodological diversity for understanding power in food policy. Each approach contributes a distinct form of insight, offering complementary ‘pieces of the jigsaw’ that together build a more comprehensive picture. Building the evidence base to collectively combine quantitative approaches with qualitative methods (Machado *et al*. 2023) or discourse analysis (Brons *et al*. 2022) and incorporate longitudinal and comparative research designs could enhance the robustness of findings. It would also capture several facets of power to provide a comprehensive understanding of power in food policy. An additional consideration in power analysis is the role of researcher reflexivity and positionality. Researchers’ own perspectives shape methodological choices, the theories of power considered relevant, and the interpretation of power dynamics (Buse *et al*. 2023). However, reflexivity and positionality were largely absent from the studies included in this review, representing an important methodological gap. Finally, many studies did not report methodological limitations or suggest areas for future research. Future studies should engage more explicitly with methodological limitations, reflexivity, and areas for further research, as this would strengthen transparency and support the development of the field.

The geographical distribution and policy focus of studies also warrants brief reflection. Whilst the number of studies across continents was relatively similar, there was a clearer concentration by income group, with most studies conducted in HICs. This pattern may reflect differences in research capacity, funding availability, and the accessibility of data in these settings, as well as the strong presence of public health nutrition and commercial determinants of health research communities in countries such as Australia and the UK. A similar concentration was observed in the policy domains examined, with most studies focused on health and agriculture policy and far fewer addressing social or environmental dimensions of food policy. Together, these patterns highlight opportunities for further empirical work in under-represented regions and policy domains.

The diversity of conceptual and analytical approaches identified in this study is consistent with previous reviews on the study of power in malnutrition policy (Walls *et al*. 2021) and food systems (Sodano and Gorgitano 2022). It was beyond the scope of this study to unpack the ontology of power explored in these studies, however, similar to the findings of Sodano and Gorgitano (2022), there was an inconsistent application of theories of power. Many studies did not provide an explicit definition of power and future research could better engage with theory and analytical tools to facilitate evidence syntheses across studies (Walls *et al*. 2021). We recommend the guide by Topp *et al*. (2021), Avelino’s (2021) paper, and the discussion of power analysis in Making Health Policy (Buse *et al*. 2023) as useful resources for selecting and applying theories of power.

This review highlighted several methodological gaps in the existing literature on power in food policy. Methods such as participatory action research and big data analytics were notably absent. Their application could offer new perspectives on power in food policy and have been previous recommended for analysing power in health policy and systems (Topp *et al*. 2021). Given the small sample of quantitative studies identified in this review, it is not possible to fully appreciate the potential utility of different quantitative methods for understanding power in food policy. Scholars keen to embrace quantitative methods could consider how different methods have been used, and to what effect, in studies at other levels of food policy or in other topic areas. For example, quantitative methods have been used to assess power in the Agricultural Committee of the European Parliament (Ferto *et al*. 2020), and local level policy (Falkowski 2017).

### Strengths and limitations of this study

This review provides guidance on methodological approaches for future research. The practical utility of this review is strengthened by the detailed critical assessment of how a range of research methods have been used to study different aspects of power and policymaking. The use of established methods for scoping reviews (Peters *et al*. 2020) and CIS (Dixon-Woods *et al*. 2006), and large number of databases included in the search represent major strengths of this study. Principal limitations of this study were the single-researcher data analysis and limited availability of a second screener. However, risk of bias for the former limitation was mitigated through regular team discussions, and the latter through the use of an established method for partial double screening (Mak and Thomas 2022).

Restrictions in the selection criteria represent an additional limitation. Power is conceptually and analytically complex (Dowding 2008). During the criteria development it became clear that including synonyms for power, such as agency, ideology, and influence, returned an unmanageably large number of results. To produce a final sample that was amenable to coherent analysis and discussion, only studies that used the word power in the title or abstract were included. It is acknowledged that this may have resulted in the exclusion of potentially relevant studies where power has not been explicitly stated, such as some ‘political economy’ studies or studies on the politics of health policy (Buse *et al*. 2023). This limitation could also have contributed to the low number of studies that assessed how less-powerful actors overcame disadvantage to influence policy, and future research is needed to map the current literature on understanding of overcoming power disparities. For similar reasons, the decision was made to limit the scope to studies in the national public policy context. Contemporary food policy involves complex governance arrangements across multiple levels of society and a wide array of actors (Feindt and Flynn 2009) that could not be fully considered in this review. Consequently, it is possible that this review missed research methods used to assess power in food policy that could be translatable to the national food policy context.

## Conclusion

This review provides insights into the wide variety of methods that have been used to investigate power in food policy. Qualitative studies dominate current research in this field, whilst discourse analytic and quantitative methods remain underutilized. Each approach offers distinct lenses that influence our understanding of power in food policy, and each have advantages and disadvantages that need to be considered. Most existing research focuses on interrogating and explaining how current power asymmetries impede progress on addressing food system challenges, with fewer studies exploring instances where less-powerful actors successfully challenged dominant interests to influence policy for the benefit of human and planetary health. This review highlights the need for methodological innovation—including greater use of mixed methods, longitudinal and comparative approaches—to address existing gaps in understanding how power manifests and is mobilized in food policymaking.

## Supplementary Material

daag104_Supplementary_Data

## Data Availability

All data are included within the manuscript and supplementary materials are available at Health Promotion International online.
