# Supplementary material for: Understanding power in food policy: a critical scoping review of methods to guide future research
Source: Health Promot Int. 2026 Jul 28;41(4):daag104. doi: 10.1093/heapro/daag104 (PMC13409316; doi:10.1093/heapro/daag104)
Supplement: daag104_Supplementary_Data [file daag104_supplementary_data.zip › Supplementary file 2 - Search strategy.docx]

**Supplementary File 2**

Complete search strategy including search strings

| Database | Search string | Parameters |
| --- | --- | --- |
| Scopus | TIT-ABS-KEY Power AND (agricult* OR nutrition* OR farm* OR diet* OR obesity OR malnutrition) AND (polic* OR politic* OR regulat* OR legislat* OR government* OR governance) | Date 2014-2024, English language, Academic article |
| Web of Science | TOPIC Power AND (food OR agri-food OR agro-food) AND (polic* OR politic* OR regulat* OR legislat* OR government* OR governance | Date 2014-2024, English language, Academic article |
| EBSCO | ABS Power AND (food OR agri-food OR agro-food) AND (polic* OR politic* OR regulat* OR legislat* OR government* OR governance) | Date 2014-2024, English language, Academic article |
| OVID Online | ABS Power AND (food OR agri-food OR agro-food) AND (polic* OR politic* OR regulat* OR legislat* OR government* OR governance) | Date 2014-2024, English language, Academic article |
| Subject / MESH term searches |  |  |
| Academic Search Ultimate | Subject Terms: (DE "FOOD and politics" AND DE "FOOD laws" AND DE "FOOD safety policy") OR (DE "NUTRITION policy") OR (DE "AGRICULTURE and politics") OR (DE "AGRICULTURAL policy") AND Power | Date 2014-2024, English language, Academic article |
| MEDLINE® | (MM“Nutrition Policy+”) AND Power  (MM*Public Policy+” OR MM“Policy Making+”) AND AB Power AND AB (food OR agri-food OR agrofood) | Date 2014-2024, English language, Academic article |
