# Supplementary material for: Understanding power in food policy: a critical scoping review of methods to guide future research
Source: Health Promot Int. 2026 Jul 28;41(4):daag104. doi: 10.1093/heapro/daag104 (PMC13409316; doi:10.1093/heapro/daag104)
Supplement: daag104_Supplementary_Data [file daag104_supplementary_data.zip › Supplementary File 3 - Included articles final.docx]

Supplementary File 3 – Included articles

| **Author(s) & year** | **Dates** | **Country** | **Policy topic** | **Object of analysis** | **Study design** | **Data collection methods and sample** | **Data analysis methods** | **Framework(s) / Tools** | **Key findings** | **Methodological Strengths** | **Methodological Limitations** |
| --- | --- | --- | --- | --- | --- | --- | --- | --- | --- | --- | --- |
| Alpha & Fouilleux (2018) | 2018 | Burkina Faso | Food security | Policy field | Qualitative | Interviews (n=54) Documents (n=?) Observation (n=?) | Qualitative analysis | Historical institutionalism Discursive institutionalism | Identifies power struggle over how food security is framed, historically dominated by productionist framing.  Linked with the historical legitimacy of agricultural actors, but challenged by ideas relating to food sovereignty, and nutrition-sensitive agriculture emerging from a push for greater intersectoral food security policies. | Utility of framework for studying institutional and beaurocratic factors in policy implementation at national and local level | None listed |
| Andersson, Pettersson & Lodin (2022) | 2022 | Rwanda | Agriculture | Policy document | Discourse analytic | Documents (n=12) | Discourse analysis | Feminist political ecology, Bacchi & Goodwin (2016) | Critically examines discursive construction of men and women in national agriculture policy documents, finding reinforcement and perpetuation of gender inequalities by constructing women farmers as problematic and separate to the normative standard applied to men - therefore fails to address structural constraints | Analysis conducted by multiple authors. | None listed |
| Attorp & McAreavey (2020) | 2020 | Northern Ireland | Agriculture | Policy document | Discourse analytic | Interviews: (n=14) Documents (n=?) | Critical discourse analysis Thematic analysis | Post-exceptionalism (Daugbjerg & Feindt 2017) | Examined the GfG strategy to identify underlying power dynamics. Despite claims for including wider interests, the GfG policy disproportionately served the poultry industry over other stakeholders - concentration of power. This impacted the perceived transparency and legitimacy of the devolved government. | None listed | None listed |
| Autzen & Hegland (2021) | 2021 | Denmark | Environment | Policy process | Qualitative Ethnography | Interviews (n=5) Documents (n=?) Observation (n=14) | Qualitative analysis Discourse analysis | Foucault Arts and van Tatenhove: structural, dispositional and relational forms of power. | Examined the power dynamics in the policy process, through the 'organisational, relational and discursive capacities' of stakeholders. Small-scale fishers were able to overcome a power disparity to influence policy | None listed | None listed |
| Babu & Akramov (2022) | 2022 | Tajikistan | Food or agri-food | Policy process | Qualitative Case study | Interviews (n=?) Documents (n=?) Focus groups (n=?) | Qualitative analysis | Stakeholder mapping framework 'veto power' (Babu 2014) | Stakeholder mapping of agricultural policy process. Finds power concentrated in central government across different development eras and structural/institutional changes. Contributed to failure of agriculture to reach its full potential. Identified a need for power sharing in participatory decision making, building capacity at the local level to deliver more effective food policies. | None listed | None listed |
| Baker et al. (2017) | 2017 | Australia | Health | Policy process | Qualitative Case study | Interviews (n=27) | Process tracing Thematic analysis | Shiffman & Smith (2007) Framework on determinants of political priority: Actor power, Ideas, Political contexts, Issue characteristics | Analysed influence of actor power on political priority and influence of different stakeholders in policy process. Structural power of food, beverage and advertising industries identified through their economic importance and access to policymakers. Employed powerful libertarian/neolibertarian rhetoric (personal responsibility, harms of regulation on the free market) to oppose regulation. Institutional norms identified as a barrier to federal government action. | Data triangulation | Single case study |
| Brookes (2021) | 2021 | UK | Health | Policy document | Discourse analytic | Documents (n=1) | Critical discourse analysis |  | The obesity policy is underpinned by “a neoliberal framework of governance and public health management” (p.2224) | Utility of CDA | Single author Single data source Subjective - selection of focus |
| Browne et al. (2021) | 2021 | Australia | Food or agri-food | Policy process | Qualitative | Documents (n=?) | Thematic analysis Content analysis Policy analysis | Political economy (interests, ideas and institutions)  Cultural identity (culture, identity and power) | Examined the representation of Aboriginal interests in policy instruments / document. Finds the exclusion of Aboriginal perspectives and organisations from the policy process reflects a colonial legacy. Both limits their power and actively "disempowers the self-determination of Aboriginal people" (p.880) | 10 year study span recommended for policy process research | 1) data: limited to publicly available formal submissions by Aboriginal actors - may miss contributions via other mechanisms, and by other actors  2) difficult to attribute influence of submissions on policy recommendations, did not assess whether policy recommendations were implemented  3) focus limited to Aboriginal participation in national level food policy, missing potential participation in other health policy or sub-national policy |
| Carey, Parker & Scrinis (2017) | 2017 | Australia | Animal welfare | Policy process | Qualitative | Documents (n=47) | Thematic analysis | Policy problem framing (Weiss, 1989) policy windows (Kingdon 2011) Advocacy Coalition Framework (Sabatier & Jenkins-Smith 1999) | Examined the power dynamics between actors in a contest over the introduction of a welfare standard for free range eggs. The initiation of standard development demonstrated civil society activism successfully challenging industry power. However, industry were able to redefine the policy problem and solution during the process, aided by the economic focus of the Regulatory Impact Statement process, which excluded environmental and welfare considerations. | None listed | None listed |
| Carriedo Lock & Hawkins (2020) | 2020 | Mexico | Health | Policy process | Qualitative Case study | Interviews (n=35) Documents (n=145) | Thematic analysis | Policy theory: Stages heuristic modes, Policy triangle framework, multiple streams model Principles of good health governance (Siddiqi et al. 2009) | Analysed power dynamics between non-state stakeholders. Identified two opposing advocacy coalitions and the strategies they used at different stages of the policy process. The dominant coalition involved the food and beverage industry who were directly involved in policy development and worked ‘as partners’ with the government. Countered by a coalition of academic and civil society actors and international organisations who sought to generate public pressure and support for the tax. | Data triangulation  Quality assurance process | Short time frame  Skewed sample: lack of participation by Market actors, reluctance of some political actors to be recorded  Positioning of the author |
| Chinsinga & Chasukwa (2018) | 2018 | Malawi | Agriculture | Policy process | Qualitative | Interviews (n=33) Documents (n=?) | Political economy analysis | Keely & Scoones (2003): narratives (knowledge and discourse, actors and networks, and politics and interests (power dynamics) | Identified underlying structural power dynamics driving dominant framing by donors. Donors’ power arises from the reliance of government agencies and NGOs on aid, resulting in competing for funds from donors. | None listed | None listed |
| Coulas (2021) | 2021 | Canada | Food or agri-food | Policy process | Qualitative Case study | Interviews (n=58) Documents (n=?) Observation (n=?) | Thematic analysis | Discursive institutionalism: power through ideas, power over ideas and power in ideas (Schmidt). Framework for Analyzing Political Discourse and Policy Change (Bhatia & Coleman 2003) | Identified how how stakeholders exerted power through ideas, power over ideas and power in ideas to influence food policy development. | None listed | None listed |
| Cullerton et al. (2016) | 2016 | Australia | Health | Policy field | Quantitative | Survey (n=140) | Social network analysis Cluster analysis | Network theory,  Advocacy Coalition Framework | Analyse relative power (as capacity) of different stakeholder to influence nutrition policy field in neoliberal country context, identifying food industry as having a distinct advantage through direct access to policymakers.  Medical professionals, and NGO / activists also had access to decision makers, although not as much or closely as industry. Academics and nutrition professionals not found to be influential. | Sample size minimises potential expansiveness bias | Defining artificial boundaries.  Skewed sample: need greater representaion from political sector  Analytic techniques: use of path distance over other measures eg. betweenness centrality  Responder bias & expansiveness bias |
| Elliott et al. (2023) | 2023 | Vanuatu | Health | Policy process | Qualitative Case study | Interviews (n=33) Documents (n=157) Observations (n=?) | Political economy analysis Thematic analysis Stakeholder analysis | Structuration theory (Giddens) Theory of capital (Boursieu) 3-I framework (ideas, interests, institutions) (Hall 1997…) Postcolonial theory | Through analysis of policy process, identified a “bidirectional relationship between structural power (ie, what is socially valued) and agentic power (ie, who influences decisions)” (p.8) Due to shifts in bureaucratic power dynamics within government, health was not a priority or interest for those with power in political agenda setting and decision-making. | Single case study: depth and richness  Data triangulation  Diverse authorship team | Single case study - limited transferability  Reliance of subjective data - perceptions of actors risks misinterpretation  Authorship team does not include a Ni-Vanuatu perspective |
| Fialon, Nabec & Julia (2022) | 2022 | Italy | Health | Policy process | Qualitative | Interviews (n=8) Documents (n=93) | Netnography Content analysis Stakeholder analysis | Stakeholder theory (Mitchell et al; Roux et al) | Analysed relative power, legitimacy and urgency of the actors involved in Nutri-Score policy process in Italy. The stakeholders who supported Nutri-Score had high legitimacy but low power.  All the stakeholders with high power and legitimacy were opposed to the policy. | Use of netnography to identify study sample  Use of established methodology for SHT | Skewed sample: no representation from industry or political parties - Perspectives of industry and parties taken from public domain to counter this |
| Fisher et al. (2021) | 2021 | Nepal | Health | Policy process | Qualitative | Interviews (n=18) Documents (n=16) | Policy analysis Thematic analysis | Baker et al. (2018) Drivers of political commitment | Examined power between stakeholders as an explanatroy variable influencing policy adoption and implementation. The primary powerholder was the government, who had the power to act/regulate as a result of a large majority. | None listed | Single author coding.  Sample skewed and limited in size.  Potential responser recall bias or social desirability bias.  Findings temporal - policy positions and conditions are ever changing |
| Georgekutty & Varghese (2024) | 2024 | India | Agriculture | Policy process | Qualitative | Documents (n=?) Observations (n=?) | Content analysis | None | Through analysis of parliamentary debates and visits to farm protests, identified how the state used ordinance-making powers reserved for emergencies to bypass parliamentary checks and balances in introducing farm bills. This ‘excessive’ use of their legal power was the catalyst for farm protests that led to changes in the legislative process. | Data triangulation.  Reflexivity: acknolwedged positionality and subjectivity.  Recognised and attempted to mitigate bias and enhance rigour. | None listed |
| Gómez-Dantes et al. (2021) | 2021 | Mexico | Health | Policy process | Qualitative | Interviews (n=18) Documents( n=?) | Stakeholder analysis Thematic analysis | PolicyMaker tool to assess power | Stakeholder analysis of power of actors involved in policy discourse. Actor power rated 'high', 'medium' or 'low'. Actors opposing an increase in the level of SSB tax were identified as having greater political power than those supporting it, with strongest opposition coming from industry. There were some government actors with high political power who opposed it, whilst others supported it. | None listed | None listed |
| Harris (2019a) | 2019 | Zambia | Health | Policy process | Qualitative | Interviews (n=71) Documents (n=?) | Thematic analysis Content analysis | Power cube (Gaventa 2006) | Found that international development organisations exercised ‘hidden’ power in policy process through the use of technical language and scientific cultures. National civil society groups were largely absent from the debate, along with individuals most affected by the issues who were also “without power” (p.136). | None listed | None listed |
| Harris (2019b) | 2019 | Zambia | Health | Policy field | Qualitative Case study | Interviews (n=71) Documents (n=?) | Thematic analysis Network analysis | Policy transfer theory Advocacy coalition network | Analysed actor power dynamics in nutrition policy . Across the two advocacy coalitions identified, the most powerful actors shown in the network map were the Cabinet, MoH, Media, Lobbyists and Donors. The food security coalition was the dominant coalition, with more government actors “the national apparatus of government”, and a long history, and 80% the government agriculture budget. Nutrition coalition was newer, and funded predominantly by international donors. | None listed | Single case study.  Netmap focus on organisations rather than individuals.  Skewed sample towards nutrition actors rather than food security |
| Henning et al. (2019) | 2019 | Ghana, Senegal, Uganda | Agriculture | Policy process | Quantitative Comparative | Survey (n=135) | Policy network analysis | Interest group theory policy networks | Analysed the informational and lobbying power of stakeholder groups in participatory policy processes in 3 countries. Distribution of power aligns with developed democratic countries.  Identified different levels of power amongst NGOs: (1) donor and research orgs were most powerful, (2) public agencies and industrial interest groups were less powerful, (3) CSO and farmer organisations were least powerful | Use of ERGM enables statistical inferences on robustness of results.   Transparency over approach to handling missing information  Robustness checks made over empirical network data - but not theoretical framework | Modelling methods requires simplification - missing other potentially important covariates.  Use of cross-sectional data and ERGM approach not capturing dynamics processes. |
| Hernandez (2017) | 2017 | Taiwan | Trade | Policy document | Qualitative Case study | Interviews (?) documents (n=?) | Qualitative analysis | Historical institutionalism | Analysis of the institutionalisation of discourses. Found that large disparities in power between stakeholders results in the adoption of the ideas of powerful actors in policy, whereas the ideas themselves gain more prominence when there is a more even distribution of power.  Examining the alignment of goals between ministries and national policy revealed institutional power asymmetries in Taiwan. | None listed | None listed |
| Kapetanaki, Tzempelikos & Halliday (2021) | 2021 | Greece | Health | Policy process | Qualitative | Interviews (n=30) Focus groups (n=59) | Thematic analysis | Relational marketing approach to actor power | Examined the impact of power (and trust and collaboration) on the development of nutrition policy. Both citizens and stakeholders (except food industry reps) perceived food industry actors as having power over government initiatives, and the promotion of economic over public health interests. Concentration of power allowed because of state weakness. Identifying a need to shift power to ensure regulation and promote greater wellbeing. | Double coded & inter-rate reliability assessed. | Skewed sample: focus groups citizens not representative of population - attempted to mitigate.  Single food policy 'pillar' focus: nutrition |
| Kinniburgh (2023) | 2023 | France | Environment | Policy process | Qualitative | Interviews (n=13) Documents (n=?) | Policy analysis | Grounded theory | Explored how expertise was constructed and mobilised to influence glyphosate policy.  Reflects underlying power structures and political interests that elevate the voices of some actors over others. | None listed | None listed |
| Larsson & Vik (2023) | 2023 | Norway | Agriculture | Policy field | Discourse analytic | Documents (n=108) | Argumentative discourse analysis (Hajer 1995) | Post-exceptionalism (Daugbjerg & Feindt 2017) | Identified dominant / hegemonic exceptionalist and post-exceptionalist storylines that dominated political discourse as an explanation for absence of meat-reduction policies | None listed | None listed |
| Madurawala et al. (2023) | 2023 | Sri Lanka | Health | Policy process | Qualitative Case study | Interviews (n=26) Documents (n=16) | Political economy analysis | Theoretical framework of ideas, institutions, power and evidence, adopted from “Kingdon’s theory of agenda-setting and Campbell’s institutionalist approach to PEA.” | Analysis of power focused the influence of particular stakeholders. Governmental actors (MoH and other depts), WHO and food industry actors identified as influential. Industry influence involved obtaining delays and revisions to the SSB tax which was considered to have negatively affected it’s effectiveness. No interference identified in implementation phase. | Multiple case studies in same context.  Data triangulation | Skewed sample: few market actors  Some documentary data unavailable |
| Mathez & Loftus (2023) | 2023 | Morocco | Agriculture | Policy document | Discourse analytic | Interviews (n=15) Documents (n=?) | Discourse analysis | Political ecology Critical theory Foucault: Power / knowledge, governmentality | Explored the "power-knowledge dynamics of the modernisation discourse” (p.87)  Revealed underlying power structures. Identified consulting companies as a powerful actor. | None listed | None listed |
| McCartan, Brimblecombe & Adams (2024) | 2024 | Australia | Food or agri-food | Policy document | Discourse analytic | Documents (n=2) | Critical discourse analysis | Fairclough’s threedimensional model of discourse (Fairclough 2010) | analysis of aboriginal plant foods policy revealed how, through the elevation of nonindigenous knowledges and interests, such policy “tacitly upholds neoliberal interests and settler colonization” (p.16). | None listed | Subjective sample selection by single researcher.   Small sample size. |
| Milsom et al. (2023) | 2023 | South Africa | Health | Policy field | Qualitative Case study | Interviews (n=25) | Participatory system dynamics modelling Purposive text analysis | Structural, instrumental, discursive power (Milsom 2020) | Modelled the political economy underpinning corporate power in NCD policy. Mapped the different mechanisms through which industry exercised structural, instrumental and discursive power over policy. | Applied validity tests for models with subset of participants | Simplification of using modelling method may have missed potentially important details.  Reliance on subjective assessments of participants: inaccuracieis, or desirability bias  Skewed sample: few high-level state actors.  Limited generalisability beyond single country context.  Potential modeler bias due to single analyst.  Preliminary analysis - needs validating. |
| Moschitz, Hrabalova & Stolze (2016) | 2016 | Czech Republic | Agriculture | Policy field | Quantitative Comparative | Interviews (n=29) | Network analysis |  | Examined the power dynamics in a policy network, identifying changes in reputational power & centrality of different actors over the 10 year period. | Study design: longitudinal comparative analysis of same policy network | None listed |
| Moyo, Thow, Florian & Drimie (2025) | 2025 | South Africa | Health | Policy field | Qualitative | Interviews (n=48) | Qualitative analysis | Instrumental, structural & discursive power (Clapp & Fuchs), Social capital (Bourdieu) | Examines the structural and discursive power dynamics in nutrition policy and governance in South Africa, identifying decision-making power of Government serving food industry interests. | None listed | None listed |
| O'Keeffe (2017) | 2017 | Australia | Agriculture | Policy document | Discourse analytic | Documents (n=39) | Discourse analysis Genealogical analysis | Governmentality (Foucault) Technologies of agency (Dean 1999) | Analysed changing constructions of farming in policy documents from state and non-state actors over time , showing how responsibility over farming has shifted from the state (in favour of deregulation) towards private investment and markets. | Long study period: 30 years | None listed |
| Phulkerd et al. (2022) | 2022 | Thailand | Health | Policy process | Qualitative | Interviews (n=20) | Thematic analysis | Fuchs' private governance framework: Structural, instrumental, discursive power (Clapp & Fuchs 2009) | Analysed perspectives of non-commercial actors on the structural, instrumental and discursive power of the food industry influencing food marketing policy in Thailand. | Qualitative interview data appropriate for rich and deep exploration | Skewed sample: few market actors  No data triangulation  Did not assess impact on policy or causal relationships |
| Pomeranz et al. (2020) | 2020 | USA | Health | Policy process | Qualitative | Documents (n=?) | Document analysis | Legal power | Examined the legal powers available to congress to institute a national fruit and vegetable subsidy scheme . Identified 3 powers available to congress to create a national F&V subsidy scheme: (1) commercial clause powers, (2) powers to tax, (3) power to spend | None listed | Sample: Potential missing relevant documents.  Focus limited to single policy option |
| Ralston, Godziewski & Carters-White (2023) | 2023 | UK | Health | Policy document | Discourse analytic | Documents (n=1) | Discourse analysis | ‘policy-as-discourse’ approach (Bacchi, 2000; Shaw, 2010) | Examined the persistence of the neoliberal paradigm in obesity policy despite a move towards more ‘upstream’ interventions. | None listed | None listed |
| Sackar et al. (2023) | 2023 | Ghana | Food or agri-food | Policy process | Qualitative Case study | Interviews (n=28) Documents (n=?) Workshop (n=?) | Thematic analysis Policy analysis | Gaventa's power cube | Examines the power structures and relations between stakeholders involved in nutrition policy process. identifying power struggles between government departments, and between state and non-state actors. | Data triangulation | Data relied on subjective assessments of participants.  Limit to documents from past 10 years may have missed relevant documents before then. |
| Sing, Mackay, Swinburn & Garton (2025) | 2025 | Chile, Canada, UK | Health | Policy process | Qualitative Multiple Case Study | Interviews (n=21) Documents (n=?) | Political economy | Not specified | Analysed how three different governments legislated unhealthy food marketing.   Identified the institutional norms and power dynamics behind policy making that impacts on how evidence-based policy making occurs, whose interests are considered. | “Comparing three cases and the political economy dimensions and power attributes has found that despite the differences in the case country and the policy outcome, there are similar dynamics at play that can influence the success of failure of a policy process.” (p.9)  Data triangulation  Using political economy theory | Small samples sizes for interviews, patchy availability of documents |
| Thomson (2017) | 2017 | Multiple | Agriculture | Policy field | Quantitative Cross-national observational | World bank dataset | Fixed-effects panel regression |  | Assessed the power of consumers and producers under different political and economic structures [democratic and authoritarian]. Greater consumer power was associated with lower agricultural support for producers under authoritarian but not democratic political regimes. Income inequality was an indicator of lower consumer power under autocracy but not in a democracy, where there was a trend towards lower producer support to reduce food prices. | Approach balances methodological rigour and meaningful results.  Attempts ot mitigate measurement error, omitted variable bias, autoregression and autocorrelation errors, and simultaneity. | Skewed sample due to low data availability in former Soviet Union, Eastern Europe and Middle East |
| Thow et al. (2021) | 2021 | Fiji | Environment | Policy process | Qualitative Case study | Interviews (n=11) | Thematic analysis Political economy analysis | Shiffman & Smith (2007) Framework on determinants of political priority: Actor power, Ideas, Political contexts, Issue characteristics | Examined influences on the policy processes of two policies. Identified power imbalances between industry and public health stakeholders as a key challenge. | Analysis of two similar policies. | Skewed sample: low industry participation  Cases from same country and field |
| Tourangeau (2017) | 2017 | Australia | Agriculture | Policy process | Discourse analytic | Documents (n=32) | Critical discourse analysis | four-dimensional understanding of power that combined Fuch’s delineation of structural, instrumental, and discursive forms of power (Fuchs 2007; Clapp & Fuchs 2009) with the concept of constitutive power (drawing from Digeser (1992), Foucault (1980, 1982), and Haugaard (2002, 2012), among others) (p.492). | Examined how power manifested in parliamentary debates. Highlighted the important but underappreciated structural and instrumental power exercised through the use of procedural tools – in this case time allocation – that limit or control the terms of debate, offering advantages for some actors over others. Normative and ideological discourse relating to neo-liberal ideas, alongside the marginalisation of food sovereignty perspectives reflect a constitutive form of power as they have pre-established influence. | Combining CDA with Fuch's framework to assess multiple dimensions of power | None listed |
| Van Lieshout et al. (2017) | 2017 | Holland | Agriculture | Policy process | Qualitative | Documents (n=?) | Qualitative analysis | Power dynamics: relational and dynamics. Pragmatist conceptualisation of power (Allen 2008). ‘power-over’, ‘power-in’, and ‘power-of’ governance processes (Torfing, Peters, Pierre, and Sorensen 2012) | Identified the actors that had ‘power-over’, ‘power-in’, and ‘power-of’, finding that including non-state actors in a deliberative policy process served to strengthen the central government’s ‘power-over’ proceedings, rather than increase the ‘power-of’ other actors. | Rigour: multiple readings of texts | None listed |
| Venegas et al. (2025) | 2025 | Australia | Health | Policy field | Qualitative | Interviews (n=24) | Qualitative analysis | Critical theory (Bronner 2002), ACF & Knowledge-to-action framework (Sabatier) | Identified examples of the structural exclusion of people with lived experience of marginalisation in policy processes and spaces.  Equalise power imbalances by listening to people with lived experience of marginalisation and elevating their power to decide and act on food policy decisions that affect them. | Diverse sample. | Limited examples of concrete attempts to create inclusive food policy. Potentially missed other voices. |
| Voigt et al. (2024) | 2024 | Austria | Agriculture | Policy field | Qualitative | Interviews (n=14) Documents (n=?) | Historical materialist policy analysis Content analysis | Structures and actors: "the systematisation of power resources by Buckel et al. (2014)... organizational, discursive, systemic resources, and strategic-structural selectivities" Food regime theory Critical state theory | Analysed power in the contestation over 'meat politics'. Different actors employ different power resources to promote their interests.  Non-dominant 'project' used discursive power to challenge dominant narrative, and were able to influence framing of animal welfare law away from prioritising economic concerns | None listed | None listed |
| Wahnschafft, Spiller & Graciano (2024) | 2024 | Argentina | Health | Policy process | Qualitative | Interviews (n=17) | Thematic analysis | Structural, instrumental, discursive power (Milsom 2020) | Examined how advocates for a public health policy leveraged structural, instrumental and discursive power to counter corporate power and promote the adoption of the policy.  Advocates accessed economic resources from philanthropic organisations to support coalition building, generated evidence, targeted advocacy to decision-makers most likely to be in favour of the policy,, and harnessed public pressure. | Data triangulation, fear of political backlash | Single case example of successful policy process. |
| Yami et al. (2019) | 2019 | Uganda | Agriculture | Policy process | Qualitative | Interviews (n=86) Focus groups (n=18x10-12) Observation (n=?) | Qualitative analysis | Structuration theory (Giddens) 'good governance' principles (Allan & Rieu-Clarke 2010) | Used stakeholder perceptions of public participation in policy processes to examine the influence of power. Found existing power relations between stakeholders constrained "the formulation of inclusive agricultural policies" (p.403). Central government actors, technical experts and donors were identified as exerting the most power over the policy process, with policy processes only progressing if there is interest from political leaders.  The lack of engagement with smallholder farmers, traditional leaders and local government leads to failure at implementation stage because the policies are not sufficiently designed to meet their needs or content with the reality at the local level. | None listed | None listed |
| Zinsli (2023) | 2023 | Ecuador | Economic | Policy process | Qualitative Case Study | Ethnographic fieldwork Interviews (n=21) Documents (n=~400) | Qualitative analysis | Grounded theory, institutional ethnography | Examined epistemic authority in the development of GI indicator, identifying the authority afforded to international experts who defined terroir in creating the GI coffee regulation. Coffee producers were able to challenge the terms of the GI based on their own authoritative knowledge. | Data triangulation.  Sample: diverse perspectives  Use of multiple "complementary analytical approaches" (p.583) | None listed |
